# Chitosan and Sodium Hyaluronate Hydrogels Supplemented with Bioglass for Bone Tissue Engineering

**DOI:** 10.3390/gels10020128

**Published:** 2024-02-05

**Authors:** Lidia Ciołek, Ewa Zaczyńska, Małgorzata Krok-Borkowicz, Monika Biernat, Elżbieta Pamuła

**Affiliations:** 1 Biomaterials Research Group, Łukasiewicz Research Network—Institute of Ceramic and Building Materials, 31-983 Krakow, Poland; 2 Hirszfeld Institute of Immunology and Experimental Therapy, Laboratory of Immunobiology, Polish Academy of Sciences, R. Weigla Str. 12, 53-114 Wroclaw, Poland; ewa.zaczynska@hirszfeld.pl; 3 Department of Biomaterials and Composites, Faculty of Materials Science and Ceramics, AGH University of Krakow, Al. Mickiewicza 30, 30-059 Krakow, Poland

**Keywords:** chitosan, hyaluronic acid, bioglass, biocomposites, cytocompatibility, pro-inflammatory cytokines

## Abstract

The aim of the study was to produce biocomposites based on chitosan and sodium hyaluronate hydrogels supplemented with bioglasses obtained under different conditions (temperature, time) and to perform an in vitro evaluation of their cytocompatibility using both indirect and direct methods. Furthermore, the release of ions from the composites and the microstructure of the biocomposites before and after incubation in simulated body fluid were assessed. Tests on extracts from bioglasses and hydrogel biocomposites were performed on A549 epithelial cells, while MG63 osteoblast-like cells were tested in direct contact with the developed biomaterials. The immune response induced by the biomaterials was also evaluated. The experiments were carried out on both unstimulated and lipopolysaccharide (LPS) endotoxin-stimulated human peripheral blood cells in the presence of extracts of the biocomposites and their components. Extracts of the materials produced do not exhibit toxic effects on A549 cells, and do not increase the production of proinflammatory cytokines tumour necrosis factor alpha (TNF-α) and interleukin (IL-6) by blood cells in vitro. In direct contact with MG63 osteoblast-like cells, biocomposites containing the reference bioglass and those containing SrO are more cytocompatible than biocomposites with ZnO-doped bioglass. Using two testing approaches, the effects both of the potentially toxic agents released and of the surface of the tested materials on the cell condition were assessed. The results pave the way for the development of highly porous hydrogel–bioglass composite scaffolds for bone tissue engineering.

## 1. Introduction

Composite biomaterials for bone tissue engineering and regeneration are evolving towards smart biomaterials [1,2]. They often contain hydrogels due to their properties, such as adequate hydration, biocompatibility, and biodegradability [3,4]. Hydrogels are used to create innovative biomaterials for tissue engineering via 3D printing [5], ensure programmed drug release, and even provide them with magnetic properties [6]. Hydrogel materials respond well to external stimuli, making them a very good choice for creating, e.g., shape memory implants [7] or injectable scaffolds [8]. Hydrogels can also be processed to form highly porous structures that are suitable for many biomedical applications [9].

The development of hydrogel biocomposites involves the addition of various refining substances, for example, hydroxyapatite ceramics, bioglass, trace element oxides, cell proliferation stimulating compounds, or compounds with antimicrobial or anti-inflammatory properties [10,11]. New biocomposites containing chitosan and bioglass have been found to be highly biocompatibile and have antimicrobial properties [12,13].

The assessment of the cytocompatibility of biocomposites for tissue regeneration can be achieved via both direct and indirect methods based on extracts prepared from the biomaterials to be tested [14,15]. Biomaterials intended to fill in bone tissue defects can cause side effects, that is, hypersensitivity, chronic inflammation, or immunosuppression/rejection of implants [16,17]. The cell immune response is critical to the induction of these effects, while assessment is considered one of the most important indicators to evaluate the systemic immune response [18,19]. This is due to the fact that the immune system plays a key role in tissue regeneration and repair [20,21]. Most of the cytokines involved in bone resorption, such as tumour necrosis factor (TNF-α), are pro-inflammatory cytokines [22] and are produced primarily by endotoxin-activated phagocytes but can also be produced by T lymphocytes and natural killer (NK) cells. Interleukin-6 (IL-6), on the other hand, is a cytokine that has a major impact on skeletal homeostasis by regulating the development of bone cell functions. IL-6 has been shown to regulate its own production (autoregulation), which is important for the bone remodelling rate [23,24,25].

Among new-generation biomaterials, hydrogel–ceramic biocomposites play an important role [26]. Biopolymers, including, for example, polysaccharides, give them flexibility and biodegradability [27,28]. Chitosan holds a special place in ongoing research [29], as it is non-toxic and biocompatible and degrades to non-toxic oligosaccharides [30]. Equally important is sodium hyaluronate, which shows multiple biological functions [31,32]. Chitosan is a natural polymer obtained via chitin deacetylation and contains linearly linked β-1,4-glycosidic bonds of D-glucosamine and N-acetyl-D-glucosamine molecules. It is biocompatible, has antibacterial and hemostatic properties, and is biodegradable [33]. Due to its osteoconductivity, it is useful in bone tissue engineering [34]. However, to enhance its mechanical properties, it is often combined with other natural polymers, ceramics, or bioglass. Hyaluronic acid is a linear polysaccharide that belongs to the glycosaminoglycan group. It interacts with various proteins and has unique physical and mechanical properties [35,36,37]. As a highly hydrophilic compound, it contributes to maintaining tissue hydration, protects cells against free radicals, and performs immunoregulatory functions. It has been used in several fields of medicine, including orthopaedics, ophthalmology, and aesthetic dermatology [37]. Ceramics, including bioglasses, on the other hand, are components of a biomaterial providing a high specific surface area, improved mechanical strength, as well as osteoconductivity, and antibacterial activity. Bioglass with the appropriate chemical composition, e.g., containing ZnO or SrO, in combination with chitosan, is a promising solution in regenerative medicine [38,39]. Zinc ions released from bioglass stimulate cell proliferation, increase bone density, and have anti-inflammatory and antibacterial effects [40,41]. Strontium ions exhibit osteoinductive effects by improving osteoblast proliferation and have antibacterial properties [42]. Furthermore, when selecting the appropriate thermal treatment parameters for bioglass, the degree of its structure and phase composition can be altered, which, as a result, affects the biocompatibility of the composite [43]. Peptide nanoparticles can also be incorporated into the composition of polysaccharide/bioglass composites [44,45], so that the beneficial effects of individual components can be maximised synergistically.

The aim of this study is to produce biocomposites made of chitosan and sodium hyaluronate hydrogels supplemented with bioglasses obtained under different conditions (temperature, time) and to perform an in vitro evaluation of their biological performance. Test methods were chosen to evaluate the possible cytotoxicity of the extracts and its influence on the immune response, as well as to study the effects of biocomposite surfaces on the bone cells in direct contact. All these phenomena, in addition to the appropriate microstructure of composite scaffolds and the bioactivity confirmed under simulated body fluid conditions, are crucial in the development of scaffolds for bone tissue engineering and the treatment of bone tissue defects.

## 2. Results and Discussion

### 2.1. Ion Release from Biocomposites

A test conducted using the inductively coupled plasma (ICP) method showed the release of Zn^2+^ and Sr^2+^ ions from selected biocomposites. The results obtained are presented in Table 1.

The concentration of Zn^2+^ ions released from the tested composites does not exceed the level considered a therapeutic dose, which, according to Kapoor [46], may be 2 mg/L. The highest concentration of Zn^2+^ ions was determined for the C–H10–P5Zn2 composite and it was equal to 0.34 ± 0.03 mg/L. The concentration of Sr^2+^ ions released from the composites is below the level considered appropriate to stimulate the osteogenic response, which, according to the literature [47], is in the range from 3 to 6 mg/L. The Sr^2+^ ion concentration closest to the lower limit of this range was determined for the C–H10–P5Sr2d sample as 2.45 ± 0.29 mg/L.

### 2.2. Bioactivity

**Figure 1:**
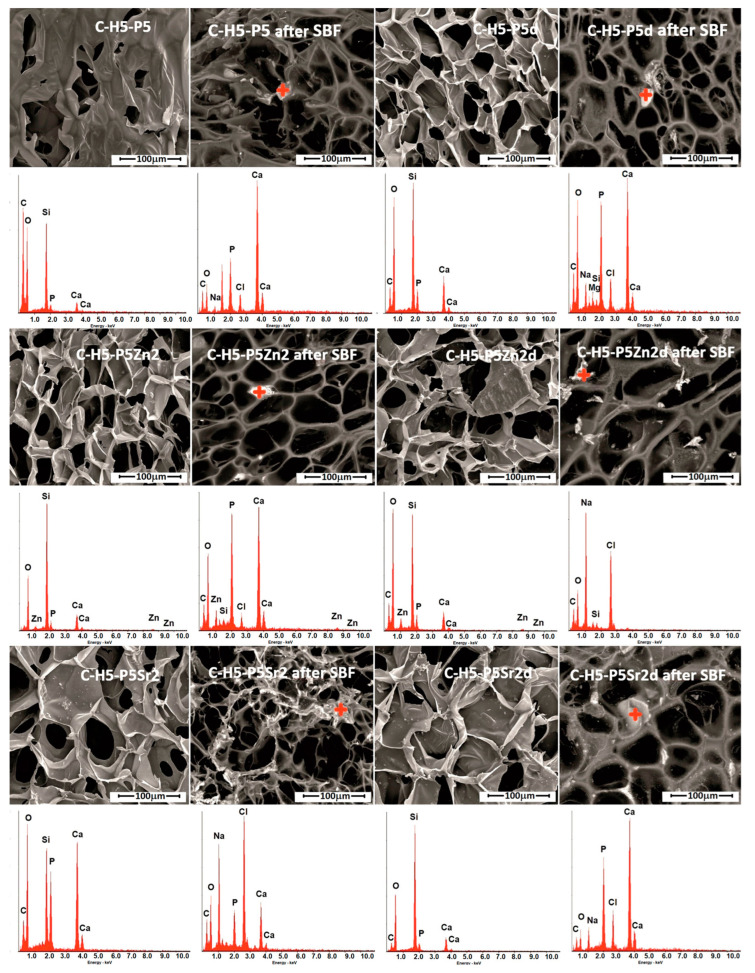
SEM-EDS analysis of chitosan/sodium hyaluronate/bioglass composites containing 5% wt. of sodium hyaluronate, 47.5% wt. of chitosan, and 47.5% wt. of bioglass treated at 550 °C/3 h (C–H5–P5, C–H5–P5Zn2, C–H5–P5Sr2) or bioglass treated at 650 °C/10 h (C–H5–P5d, C–H5–P5Zn2d, C–H5–P5Sr2d) before and after 21 days of incubation in SBF solution. Scale bar in SEM pictures is equal to 100 µm.

The bioactivity potential of the produced composites was evaluated after incubation in simulated body fluid (SBF) at 37 °C for 21 days. The SEM images and the results of qualitative chemical composition studied by means of EDS analysis before and after the incubation of the composites in SBF are shown in Figure 1. An EDS analysis conducted near the bioglass grains after the incubation of the prepared samples in SBF demonstrated a decrease in the Si peak and an increase in the intensity of the Ca and P peaks, suggesting the formation of calcium phosphates on their surface, as shown earlier [38,48]. The SEM observation results for the biocomposites show that these are highly porous, with an average pore size of 100 µm. The results of the test conducted in SBF show that chitosan-sodium-hyaluronate–bioglass biocomposites exhibit the ability to form apatite on the surface when exposed to SBF solution, and thus can be regarded as bioactive.

### 2.3. Cytotoxicity Testing of Biocomposites against A549 Cells

**Figure 2:**
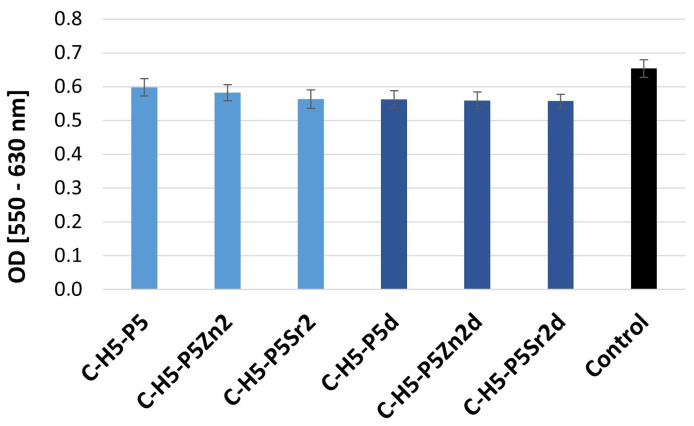
Viability of A549 cells (assessed via MTT test) cultured in extracts from composites containing 5% wt. of sodium hyaluronate, 47.5% wt. of chitosan, and 47.5% wt. of bioglass treated at 550 °C/3 h (C–H5-P5, C–H5–P5Zn2, C–H5–P5Sr2) or bioglass treated at 650 °C/10 h (C–H5–P5d, C–H5–P5Zn2d, C–H5–P5Sr2d).

**Figure 3:**
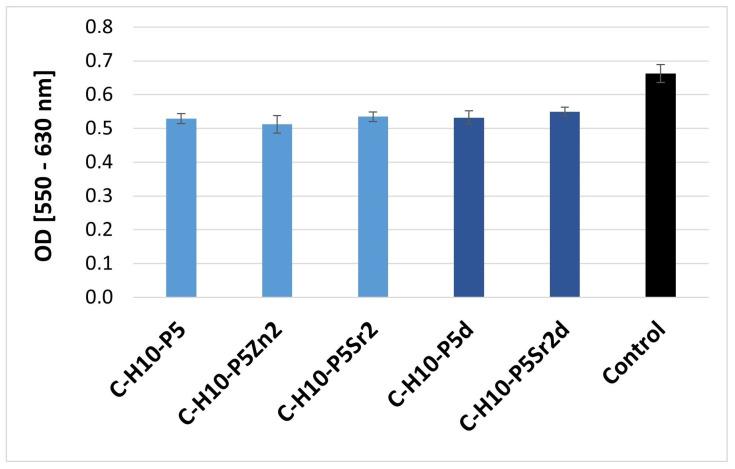
Viability of A549 cells (assessed via MTT test) cultured in extracts from composites containing 10% wt. of sodium hyaluronate, 45% wt. of chitosan, and 45% wt. of bioglass treated at 550 °C/3 h (C–H10–P5, C–H10–P5Zn2, C–H10–P5Sr2) or bioglass treated at 650 °C/10 h (C–H10–P5d, C–H10–P5Sr2d).

The cell growth, viability, and cell morphology were examined as parameters determining the potential cytotoxicity of the biomaterials. The conditions of the A549 cell culture in contact with extracts of the composites were assessed using an indirect method. The cytotoxicity of the tested extracts was determined by measuring the viability of the A549 cells. The results are shown as mean optical density (OD) values from six wells ± standard deviation (±SD) (Figure 2 and Figure 3).

The cell viability was measured via the precise and fast colorimetric MTT test [49]. Cells were exposed to extracts at specific concentrations for 48 h. The results show that the cell viability was not influenced by the presence of all the samples containing 5% wt. (Figure 2) or 10% wt. sodium hyaluronate (Figure 3): the decrease in OD was less than 70%, which, according to ISO10993-5 [50], means that the material is not cytotoxic.

In the results presented, no significant differences between the samples were recorded. No cell damage was observed; all cells had a normal morphology and good proliferation compared to the control conditions.

### 2.4. The Effect of Extracts on Cytokine Production in Cell Cultures

**Figure 4:**
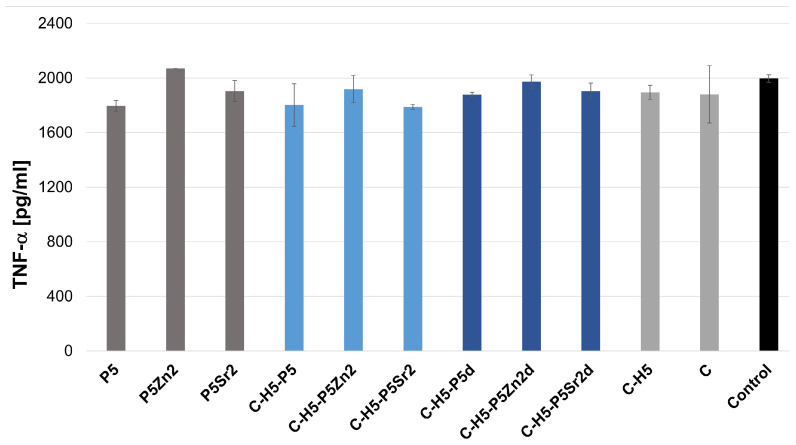
Production of LPS-induced TNF-α in WBC cultures in the presence of extracts from bioglass samples treated at 550 °C/3 h (P5, P5Zn2, P5Sr2) and composites containing 5% wt. of sodium hyaluronate, 47.5% wt. of chitosan, and 47.5% wt. of bioglass treated at 550 °C/3 h (C–H5–P5, C–H5–P5Zn2, C–H5–P5Sr2) or at 650 °C/10 h (C–H5–P5d, C–H5–P5Zn2d, C–H5–P5Sr2d) and the reference: hydrogel containing 95% wt. chitosan and 5% wt. sodium hyaluronate (C–H5) and hydrogel containing 100% wt. chitosan (C) in comparison with WBC culture without any extract (control).

**Figure 5:**
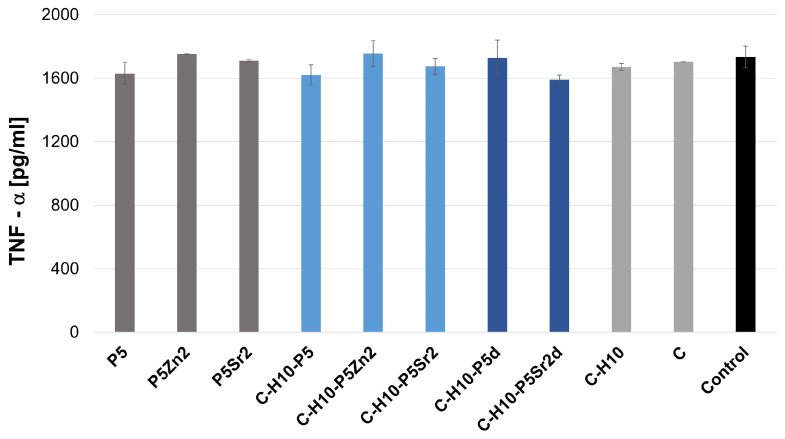
Production of LPS-induced TNF-α in WBC cultures in the presence of extracts from glass samples treated at 550 °C/3 h (P5, P5Zn2, P5Sr2) and composites containing 10% wt. of sodium hyaluronate, 45% wt. of chitosan, and 45% wt. of bioglass treated at 550 °C/3 h (C–H10–P5, C–H10–P5Zn2, C–H10–P5Sr2) or at 650 °C/10 h (C–H10–P5d and C–H10–P5Sr2d) and the reference: hydrogel containing 95% wt. chitosan and 5% wt. sodium hyaluronate (C–H5) and hydrogel containing 100% wt. chitosan (C) in comparison with WBC culture without any extract (control).

Figure 4 and Figure 5 show the effect of the extracts of the biocomposites and their components on LPS-induced TNF-α production in a whole blood culture (WBC)**.** Using an ELISA test, no spontaneous production of TNF-α and IL-6 by human blood cell cultures, activated with biocomposite extracts alone, was demonstrated, as the detection limit of the TNF-α and IL-6 assay is 2 pg/mL and 4 pg/mL, respectively. WBC exposed to extracts from composites and their components, i.e., bioglasses, chitosan, and chitosan–hyaluronic-acid mixtures with the addition of LPS, did not show any altered production of the tested cytokines compared to the level of the tested cytokines determined in a culture with LPS only (control). No significant differences were found between any of the samples according to ANOVA.

**Figure 6:**
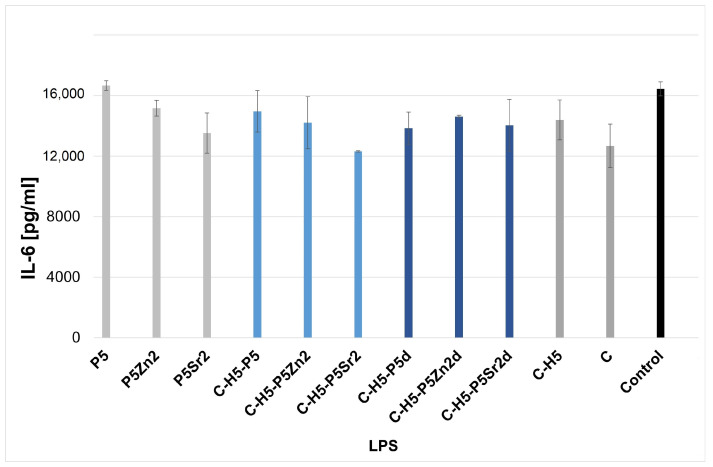
Production of LPS-induced IL-6 in WBC cultures in the presence of extracts from glass samples treated at 550 °C/3 h (P5, P5Zn2, P5Sr2) and composites containing 5% wt. of sodium hyaluronate, 47.5% wt. of chitosan, and 47.5% wt. of bioglass treated at 550 °C/3 h (C–H5–P5, C–H5–P5Zn2, C–H5–P5Sr2) or at 650 °C/10 h (C–H5–P5d, C–H5–P5Zn2d, C–H5–P5Sr2d) and the reference: hydrogel containing 95% wt. chitosan and 5% wt. sodium hyaluronate (C–H5) and hydrogel containing 100% wt. chitosan (C) in comparison with WBC culture without any extract (control).

**Figure 7:**
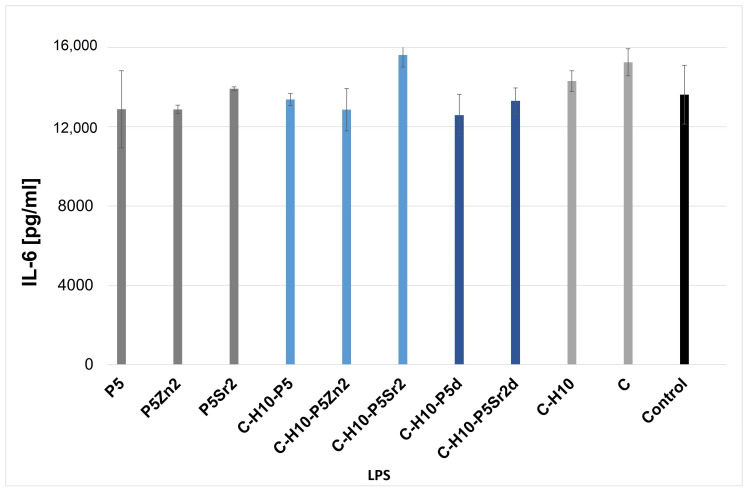
Production of LPS-induced IL-6 in WBC cultures in the presence of extracts from glass samples treated at 550 °C/3 h (P5, P5Zn2, P5Sr2) and composites containing 10% wt. of sodium hyaluronate, 45% wt. of chitosan, and 45% wt. of bioglass treated at 550 °C/3 h (C–H10–P5, C–H10–P5Zn2, C–H10–P5Sr2) or at 650 °C/10 h (C–H10–P5d and C–H10–P5Sr2d) and the reference: hydrogel containing 90% wt. chitosan and 10% wt. sodium hyaluronate (C–H10) and hydrogel containing 100% wt. chitosan (C) in comparison with WBC culture without any extract (control).

Figure 6 and Figure 7 show the effect of the extracts from the biocomposites and reference samples on LPS-induced IL-6 production in a WBC culture. No significant differences were found between all the samples according to ANOVA.

TNF-α plays a fundamental role in the regulation of bone homeostasis in various diseases. The impact of this cytokine can be classic or paradoxical [51]. Our results are consistent with the results obtained by Osto [51] and Huang [52], who demonstrated that TNF-α released at levels of 0.01–0.1 ng/mL induces osteogenic differentiation, while, after exposure to LPS, the amounts of TNF-α fall within the range of 1–20 ng/mL, which, according to the literature [53,54], inhibits preosteoblast differentiation. The literature also indicates that TNF-α may be a cytokine that initiates tissue healing [55].

IL-6, on the other hand, regulates the differentiation of both osteoblasts and osteoclasts, but its impact is limited to the early stages of fracture healing [53]. Figure 6 and Figure 7 show the effect of composite extracts and their components on LPS-induced IL-6 production in WBC cultures. Human peripheral WBC cultures exposed to LPS and extracts of the biocomposites tested produced IL-6 at the same level as cytokines secreted by blood cells only with LPS. Exposure to all the extracts tested did not result in a significant release of these pro-inflammatory cytokines, regardless of the chemical and phase composition of the bioglass and the proportion of the components in the composites. This can only be noted when compared to the reference and SrO-doped bioglasses and composites containing this bioglass. In this case, the amount of IL-6 seems to be slightly higher, but the difference is not statistically significant. The extracts of the tested materials have been shown to not affect the production of pro-inflammatory mediators by human blood cells. After stimulation with LPS endotoxin and extracts, the level of cytokines released was at the same level as for the control, i.e., LPS-stimulated blood cells. LPS strongly stimulates the immune response [56]. Lastly, no statistical differences were found between the extracts of the composites tested. Thus, the results show that the leached composite components do not affect the in vitro release of the pro-inflammatory cytokines known to be involved in bone resorption associated with, for example, the aseptic loosening of the implant.

### 2.5. Cytocompatibility with MG63

**Figure 8:**
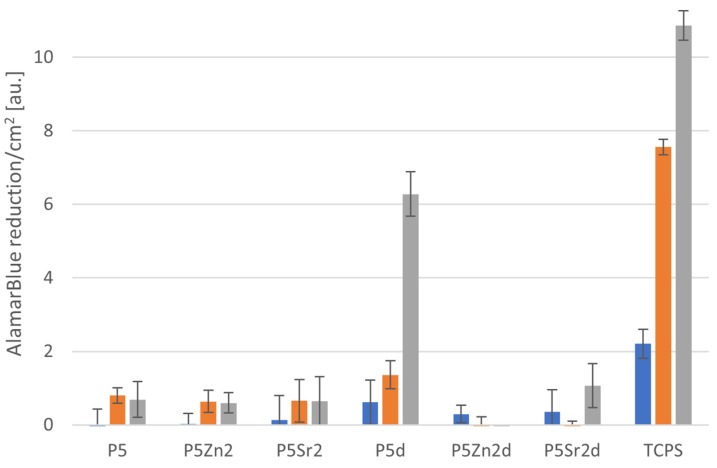
Viability of MG-63 osteoblast-like cells cultured on bioglasses (P5, P5Zn2, P5Sr2 and P5d, P5Zn2d, P5Sr2d) tested using AlamarBlue^®^ on days 1, 3, and 7 (blue, yellow, and grey bars, respectively). The results are presented as average standard deviation, n = 3; statistically significant differences compared to TCPS for the corresponding time points at *p* < 0.001 were found for all the samples.

**Figure 9:**
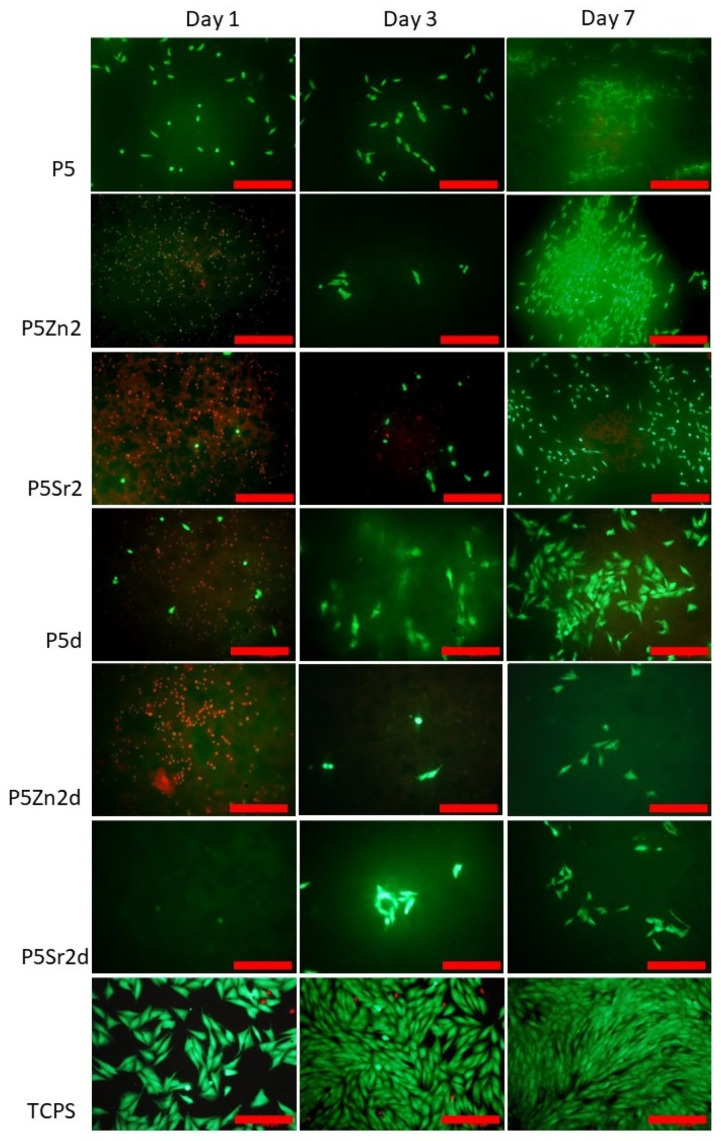
Live/dead viability staining (green—live cells; red—dead cells) for MG63 cells cultured on bioglasses (P5, P5Zn2, P5Sr2 and P5d, P5Zn2d, P5Sr2d) on days 1, 3, and 7. Scale bar—200 μm.

**Figure 10:**
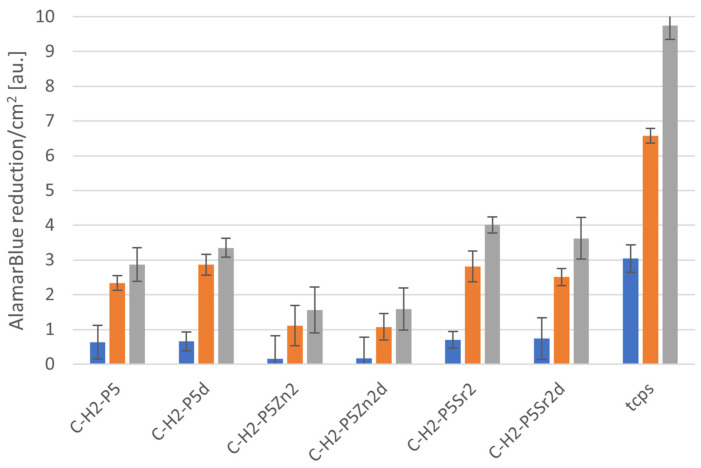
Viability of MG-63 osteoblast-like cells cultured on biocomposites containing 2% wt. of sodium hyaluronate, 49% wt. of chitosan, and 49% wt. of bioglass treated at 550 °C/3 h (C–H2–P5, C–H2–P5Zn2, C–H2–P5Sr2) or at 650 °C/10 h (C–H2–P5d, C–H2–P5Zn2d, C–H2–P5Sr2d) tested using AlamarBlue^®^ on days 1, 3, and 7 (blue, yellow, and grey bars, respectively). The results are presented as average standard deviation, n = 3; statistically significant differences compared to TCPS for the corresponding time points at *p* < 0.001 were found for all the samples.

**Figure 11:**
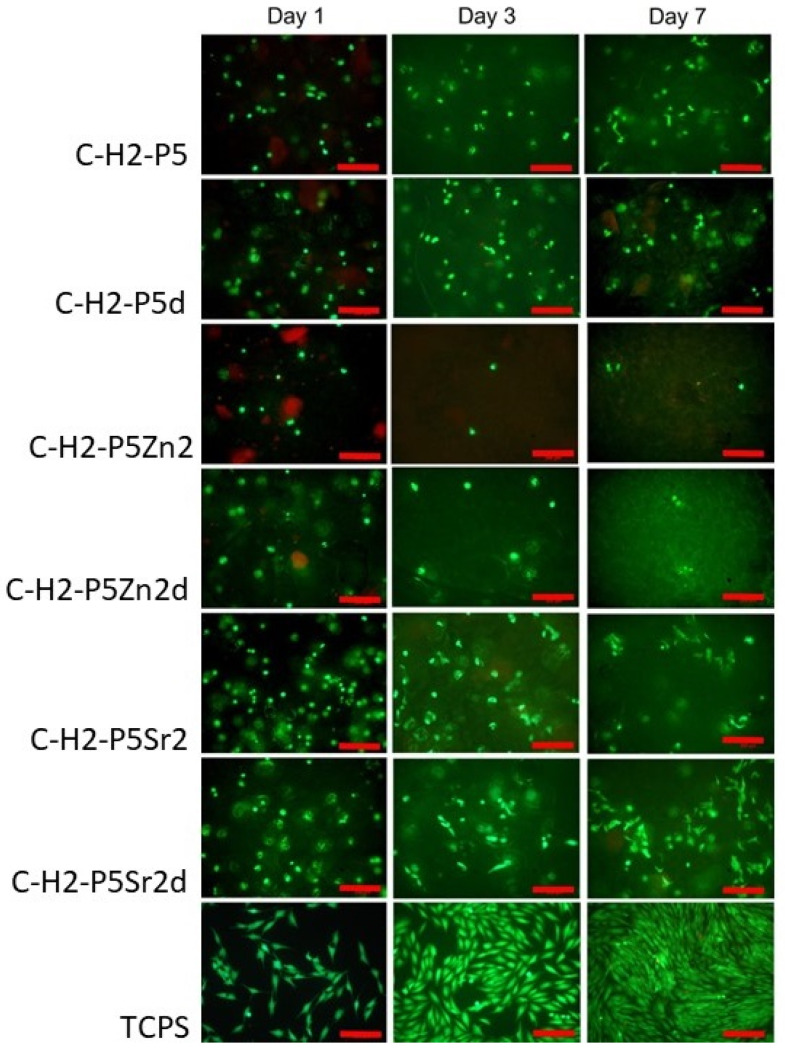
Live/dead viability staining (green—live cells; red—dead cells) for MG63 cells cultured on biocomposites (containing 2% wt. of sodium hyaluronate, 49% wt. of chitosan, and 49% wt. of bioglass treated at 550 °C/3 h (C–H2–P5, C–H2–P5Zn2, C–H2–P5Sr2) or at 650 °C/10 h (C–H2–P5d, C–H2–P5Zn2d, C–H2–P5Sr2d) on days 1, 3, and 7. Scale bar—200 μm.

**Figure 12:**
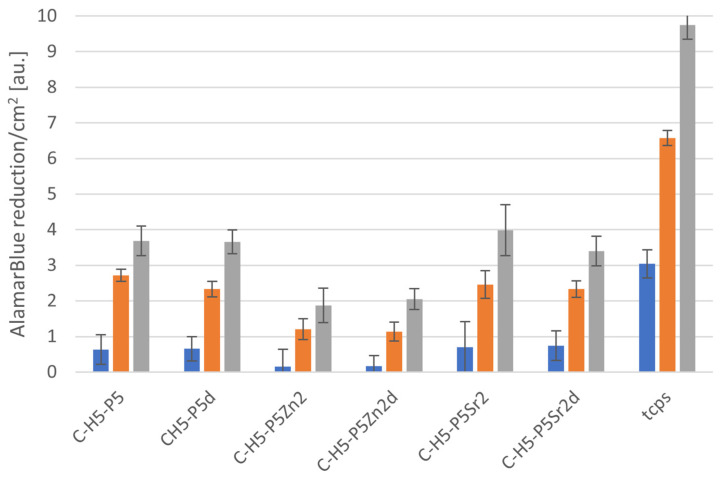
Viability of MG-63 osteoblast-like cells cultured on biocomposites containing 5% wt. of sodium hyaluronate, 47.5% wt. of chitosan, and 47.5% wt. of bioglass treated at 550 °C/3 h (C–H5–P5, C–H5–P5Zn2, C-H5-P5Sr2) or at 650 °C/10 h (C–H5–P5d, C–H5–P5Zn2d, C–H5–P5Sr2d) (tested using AlamarBlue^®^ on days 1, 3, and 7 (blue, yellow, and grey bars, respectively). The results presented as average standard deviation, n = 3; statistically significant differences compared to TCPS for the corresponding time points at *p* < 0.001 were found for all the samples.

**Figure 13:**
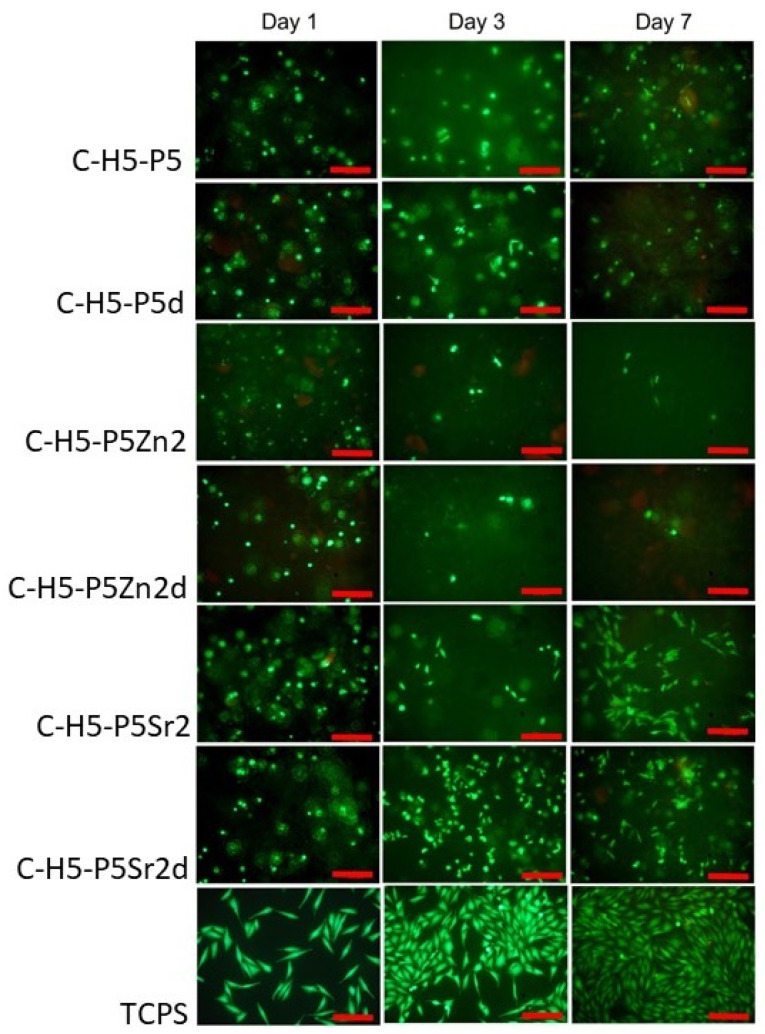
Live/dead viability staining (green—live cells; red—dead cells) for MG63 cells cultured on biocomposites containing 5% wt. of sodium hyaluronate, 47.5% wt. of chitosan, and 47.5% wt. of bioglass treated at 550 °C/3 h (C–H5–P5, C–H5–P5Zn2, C–H5–P5Sr2) or at 650 °C/10 h (C–H5–P5d, C–H5–P5Zn2d, C–H5–P5Sr2d) on days 1, 3, and 7. Scale bar—200 μm.

**Figure 14:**
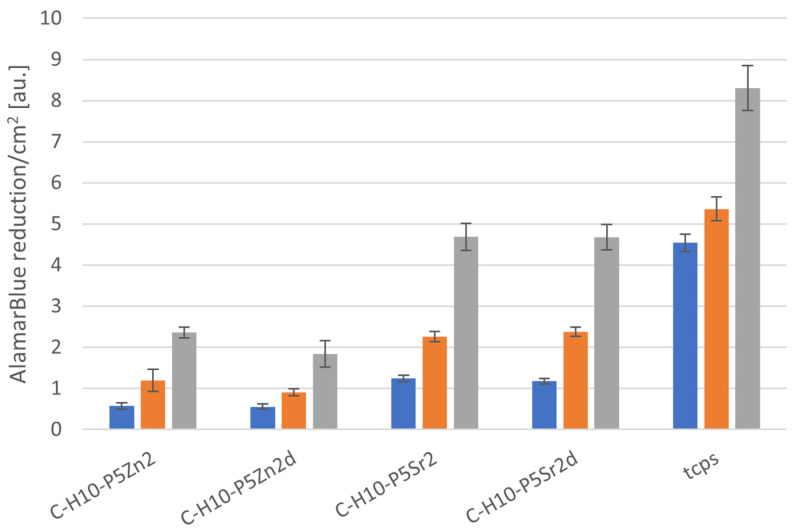
Viability of MG-63 osteoblast-like cells cultured on biocomposites containing 10% wt. of sodium hyaluronate, 45% wt. of chitosan, and 45% wt. of bioglass treated at 550 °C/3 h (C–H5–P5, C–H5–P5Zn2, C–H5–P5Sr2) or at 650 °C/10 h (C–H5–P5d, C–H5–P5Zn2d, C–H5–P5Sr2d) (tested using AlamarBlue^®^ on days 1, 3, and 7 (blue, yellow, and grey bars, respectively). The results are presented as average standard deviation, n = 3; statistically significant differences compared to TCPS for the corresponding time points at *p* < 0.001 were found for all the samples.

**Figure 15:**
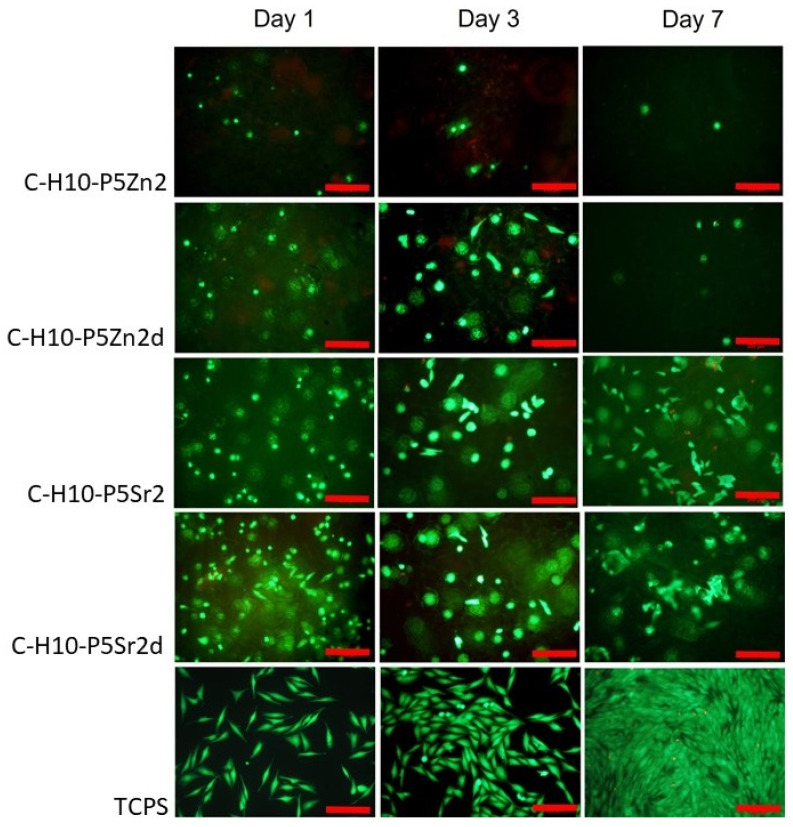
Live/dead viability staining (green—live cells; red—dead cells) for MG63 cells cultured on biocomposites containing 10% wt. of sodium hyaluronate, 45% wt. of chitosan, and 45% wt. of bioglass treated at 550 °C/3 h (C–H5–P5, C–H5–P5Zn2, C–H5–P5Sr2) or at 650 °C/10 h (C–H5–P5d, C–H5–P5Zn2d, C–H5–P5Sr2d) on days 1, 3, and 7. Scale bar—200 μm.

All bioglasses and biocomposites obtained with the bioglasses after thermal treatment at 550 °C/3 h or 650 °C/10 h were tested in vitro in direct contact with MG63 cells. The results are presented in Figure 8 and Figure 9 for bioglasses and in Figure 10, Figure 11, Figure 12, Figure 13, Figure 14 and Figure 15 for biocomposites. The results show that after 1 day of direct contact, the bioglasses were toxic to MG63 cells. The observed effect may be influenced by the ions released from the bioglasses (Ca^2+^, Zn^2+^, Sr^2+^), which increase the pH of the microenvironment [57]. After 7 days of culture, the surviving cells readily proliferated, especially when exposed to the P5, P5Zn2, P5Sr2, and P5d bioglasses (Figure 9). Composites with the reference bioglass or doped with SrO were more cytocompatible with MG63 osteoblasts than biocomposites with bioglass doped with ZnO. Bioglasses enriched with ZnO are well known to have antibacterial properties, due to the released zinc ions (Zn^2+^), which have been identified to exhibit antimicrobial activity against various bacterial and fungal strains [58]. In addition to the activity of soluble zinc species, there are additional mechanisms of antimicrobial activity: the generation of reactive oxygen species and the direct contact of the materials containing ZnO with cells walls [59]. All of these phenomena may cause cytotoxicity in eucaryotic and mammalian cells [60]. Thus, it is of key importance to adjust the concentration of zinc in bioglasses in the resulting composites in order to provide the material with antibacterial properties but not negatively impact cell adhesion and growth through direct contact.

Biocomposites with SrO-doped bioglass did not show improved cell viability compared to biocomposites with the reference bioglass, although they released Sr^2+^ ions at a level that was convenient to stimulate osteoinductivity [47,61].

The positive effect of the bioglass annealing conditions is evident only in the case of the P5d bioglass. The cell metabolic activity increased at each cell culture time point; however, for 1 day of culture, it was always lower than in the control conditions (TCPS). The live/dead staining of all the materials studied confirmed the AlamarBlue^®^ viability results. The cells exhibited a spread polygonal morphology, which was similar to that of those growing in TCPS (Figure 9, Figure 11, Figure 13 and Figure 15). The composites with Sr-containing bioglass did not show enhanced cell viability compared to the base bioglass, although they released Sr^2+^ ions.

**Figure 16:**
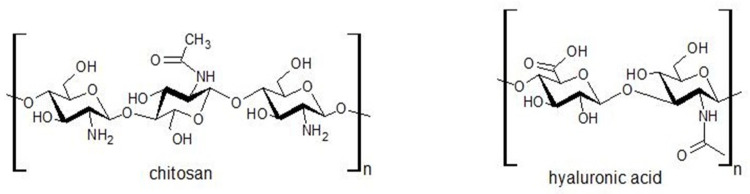
Chemical structures of chitosan and hyaluronic acid.

After analysing the obtained results for the direct cell culture on bioglasses and biocomposites, it can be concluded that the proliferation and survival of cells in contact with the composites are mainly affected by the chemical composition of the introduced bioglasses. There was no significant effect of bioglass thermal treatment or the amount of sodium hyaluronate introduced into the biocomposite matrix although, according to the literature, sodium hyaluronate increases the cell proliferation rate [62] and promotes cell migration [63]. In addition, both metabolic activity tests and live/dead staining of MG63 cells confirm that in the chitosan/sodium hyaluronate and bioglass composites tested, the surface condition plays a major role, as the release of toxicity from the ions is protected by the organic matrix in the composites, compared to the results obtained using the indirect method. The functional groups ( OH, NH_2_, >C=O) present in chitosan (Figure 16) can interact with the amide or hydroxyl groups present in sodium hyaluronate, hydrogen bonds may be formed, or electrostatic interactions may occur. These interactions between the components of the mixture lead to changes in homogeneity, which may result in surface roughness. According to Lewandowska et al. [64], the structure of the chitosan and hyaluronic acid mixtures depends on its composition and the type of solvent used. The results are in line with the evaluation conducted by Denuziere, who demonstrated that chitosan in contact with cells gives better outcomes than chitosan–chondroitin-sulfate and chitosan–hyaluronate-polyelectrolyte complexes, mainly because of the surface smoothness and electrostatic charges. In complexes, the positively charged smooth surface of chitosan is neutralised, which adversely affects the cell adhesion [65]. The results obtained indicate the need to modify the surface of the produced composites in order to improve cell adhesion, e.g., by functionalisation with RGD peptides [66,67].

## 3. Conclusions

In conclusion, the results have shown that the extracts of the tested biocomposites, differing in terms of the amount of chitosan and sodium hyaluronate hydrogels as well as bioglass with varied chemical compositions and thermal treatment parameters, are not cytotoxic for the A549 cells in indirect contact. They also do not enhance the production of TNF-α and IL-6 by human peripheral WBC cultures. However, in direct contact with MG63 osteoblast-like cells, bioglasses are more toxic than composites. Among composites, those with the reference bioglass or SrO-doped bioglass are more cytocompatible than composites with ZnO-doped bioglass. By using two methods of cytotoxicity testing, the impact on cell conditions was evaluated for both released the toxic agents and the surface of the tested materials. The proposed research methodology also allows a complete assessment of the cytotoxicity/cytocompatibility and immune responses induced by the biomaterial, which are critical to bone regeneration and play a vital role in the initial period after the biomaterial is implanted.

To summarise, it was found that highly porous composite scaffolds based on bioglasses and polysaccharide hydrogels (1) were found microstructurally stable and bioactive according to the simulated body fluid test, (2) were able to release ions such as Sr^2+^ and Zn^2+^, which are of biological relevance, and (3) are not cytotoxic, as shown via indirect tests.

Interestingly, biocomposites containing reference bioglass or SrO-doped bioglass are more cytocompatible than ZnO-doped bioglass, which suggests that the composition of the latter sample should be optimised in further studies.

## 4. Materials and Methods

### 4.1. Materials and Chemicals

To obtain bioglass and chitosan/sodium hyaluronate/bioglass composites, the following components were used: tetraethoxysilane (TEOS); calcium nitrate (V) tetrahydrate; zinc nitrate (V) hexahydrate, ethanol 96% p.a. grade; 80% pure acetic acid (Avantor Performance Materials Poland S.A., Gliwice, Poland); triethyl phosphate (V) (Fluka, Chemie GmbH, Buchs, Switzerland); strontium nitrate (V) (Sigma-Aldrich, St. Louis, MO, USA); chitosan of a 75% deacetylation degree and 500 mPas viscosity (HMC+—Heppe Medical Chitosan GmbH company, Halle, Germany); and Sodium Hyaluronate 90 130 kDa (Contipro a.s., Dolni Dobrouc, Czech Republic) (chemical structure shown in Figure 16).

RPMI-1640 medium was purchased from Biowest (Nuaillé, France). Foetal calf serum (FCS) was purchased from HyClone (Pittsburgh, PA, USA). Other reagents such as L-glutamine, penicillin, and streptomycin solution, lipopolysaccharide (LPS) of *Escherichia coli* strain O111:B4 (L4130), and MTT (3-[4,5-dimethylthiazol-2-yl]-2,5-diphenyltetrazoliumbromide) were purchased from Sigma-Aldrich (St. Louis, MO, USA). TNF-α and IL-6 levels in the supernatants were determined using an ELISA kit from Biolegend.

The human lung adenocarcinoma cell line A549 (ATCC CCL 185) was derived from the Institute of Immunology and Experimental Therapy collection of cell lines (Wrocław, Poland), while MG63 cells (human osteoblast-like MG63 cells) were from the European Collection of Cell Cultures, Salisbury, UK. A549 cells were cultured in RPMI-1640 medium with 100 U/mL penicillin, 100 µg/mL streptomycin, 2 mM L-glutamine, and 10% FCS.

### 4.2. Preparation of Sol–Gel-Derived Bioglasses

The chemical compositions of the bioglasses (P5, P5Zn2, and P5Sr2) were developed in the SiO_2_–P_2_O_5_–CaO–ZnO–SrO system. The reference material (bioglass P5) constituted 70 wt.% SiO_2_, 5 wt.% P_2_O_5_, and 25 wt.% CaO. In the chemical compositions of the P5Zn2 bioglass, 2 wt.% CaO was replaced with ZnO and, in the P5Sr2 bioglass, 2 wt.% CaO was replaced with SrO. These bioglasses were produced using the low-temperature sol–gel method. After being converted from sols to dry gels, the reaction mixtures were dried. Subsequently, each portion of dry gel was divided into five parts. The obtained samples were subjected to thermal treatment in an electric furnace. The thermal treatment parameters of the all samples comprised annealing at 550 °C for 3 h (550 °C/3 h) or annealing at 650 °C for 10 h (650 °C/10 h). After thermal treatment, the powders were ground in a mechanical mortar for 15 min. Powders with dv (0.5) characteristic values ranging from 47 to 66 µm were obtained. Particle size analysis was performed using a Malvern Instruments 2000 laser analyser. The designed value, dv (0.5), means that the size in 50% *v*/*v* of particles in the sample population is smaller than the value dv. The phase compositions of bioglass post thermal treatment are presented in our previous publication [43]. In bioglass annealed at 650 °C/10 h, a significantly higher crystalline phase content was observed compared to bioglass annealed at 550 °C/3 h. In brief, for P5, bioglass crystallinity increased from 41.2 to 44.8 wt.%; for P5Sr2, it increased from 22.0 to 24.4 wt.%; but for P5Zn2, it practically did not change (24.2 and 24.3 wt.%) [42]. The following crystalline phases were identified in the reference and SrO-doped bioglass: pseudowollastonite, wollastonite, and quartz. In contrast, hydrastonite and crystobalite were further identified in ZnO-doped bioglass [43]. Finally, the bioglass samples were used as components of the chitosan/sodium hyaluronate/bioglass composites (Table 2).

### 4.3. Preparation of Chitosan and Sodium Hyaluronate Hydrogels Supplemented with Bioglass

**Figure 17:**
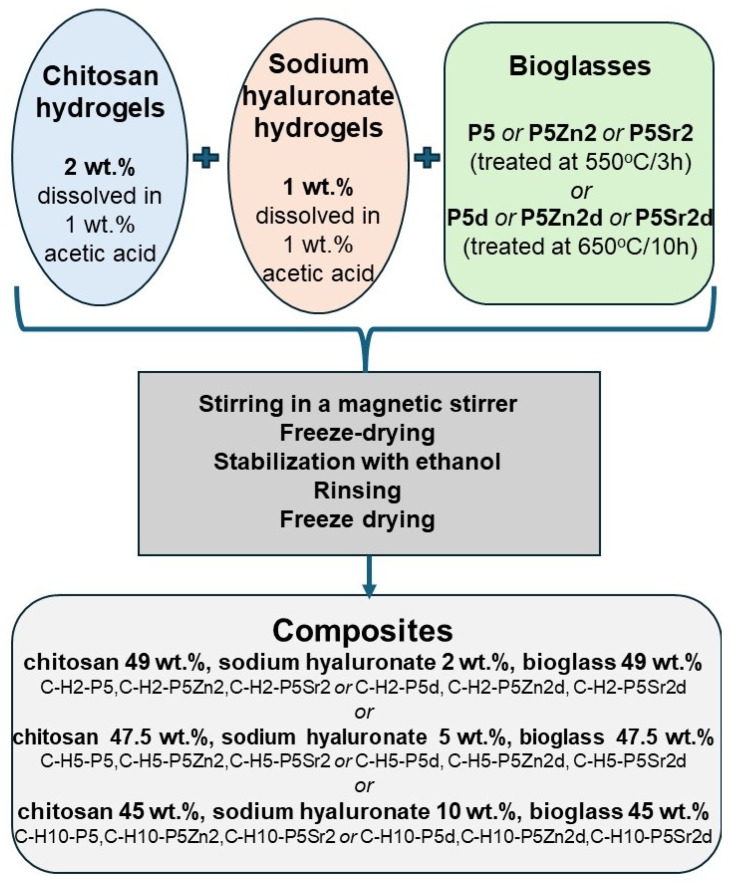
Scheme of producing chitosan-sodium-hyaluronate–bioglass composites.

Chitosan hydrogels with a concentration of 2 wt.% were prepared via dissolving in 1 wt.% acetic acid. Sodium hyaluronate hydrogels with a concentration of 1 wt. % were prepared via dissolving in 1 wt.% acetic acid. The bioglass particles were mixed with chitosan, maintaining a 1:1 weight ratio of bioglass to chitosan and with 2 wt.%, 5 wt.%, or 10 wt.% sodium hyaluronate. The C-H2 composites contain 49 wt.% chitosan, 49 wt.% bioglass, and 2 wt.% sodium hyaluronate. The C-H5 composites contain 47.5 wt.% chitosan, 47.5 wt.% bioglass, and 5 wt.% sodium hyaluronate. The C-H10 composites contain 45 wt.% chitosan, 45 wt.% bioglass, and 10 wt.% sodium hyaluronate. Hydrogels and glass particles were mixed using a magnetic stirrer. The composites were obtained by freeze-drying these mixtures. Freeze drying was carried out for 28 h (2-16D Epsilon freeze-dryer, Christ). After drying, the samples were immersed in ethanol for 6 h. Then, they were rinsed four times with deionised water, frozen to −35 °C, and again freeze-dried (Figure 17). Eighteen chitosan/sodium hyaluronate/bioglass composites were obtained (Table 3).

### 4.4. Particle Size Analysis

The analysis was performed using a laser particle size analyser (Malvern Instruments 2000, Malvern Instruments Ltd., Malvern, United Kingdom), using the low-angle laser-light-scattering (LALLS) method. The method used allowed the assessment of particle size to be carried out in the wide range of 0.1 μm–2000 μm with a margin error of 0.5%. The following characteristic values were determined: dv (0.1), dv (0.5), and dv (0.9), which means that the size of 10% *v*/*v*, 50 *v*/*v*%, or 90 *v*/*v*% of particles in the sample population is smaller than the value dv.

### 4.5. Inductively Coupled Plasma Method

Composites with 10 wt.% sodium hyaluronate supplemented with bioglass doped with SrO or ZnO, treated at 550 °C/3 h and 650 °C/10 h, were tested for ion release (Sr^2+^, Zn^2+^); 0.2 g of composite was weighed and transferred to a glass vessel, immersed in 100 mL of deionised water, and sealed. The conductivity of deionised water was 0.5 μS/cm. The samples were conditioned at a constant temperature of (37 ± 1) °C for 24 h and 7 days. The concentration of released zinc and strontium ions was determined using the inductively coupled plasma method (ICP-EOS, iCAP PRO XP; Thermo Fisher Scientific, Waltham, MA, USA). A coverage factor of k = 2 and a confidence level of 95% were used for the determinations.

### 4.6. Microstructure and Bioactivity Analysis

A simulated body fluid (SBF) with the chemical composition of blood plasma was prepared by dissolving NaCl, NaHCO_3_, KCl, K_2_HPO_4_, MgCl_2_∙6H_2_O, CaCl_2_, Na_2_SO_4_, and (CH_2_OH)_3_CNH_3_ in deionised water, according to the Kokubo procedure. The pH of the SBF solution was established at 7.25 with the use of 2 M HCl. Biocomposite samples (diameter of 12 mm and a height of 2 mm) were prepared by casting in PTFE moulds.

Six discs of each composite were placed in a glass vessel, poured with 65 mL of SBF solution, and tightly sealed. The sample vessels were incubated at a temperature close to the temperature of the human body (37 ± 1) °C for up to 3 weeks. After 21 days of incubation, the samples were removed, rinsed with deionised water, dried, and subjected to scanning electron microscopy (Nova NanoSEM 200, FEI) with energy dispersion spectroscopy (EDS) analyses. Before incubation in SBF SEM, pictures were taken using an ETD detector, while, after incubation in SBF, we better visualised microstructural changes using a vCD detector.

### 4.7. Cytotoxicity Test with A549 Cells via Indirect Method

The evaluation involved composites containing 5 wt.% sodium hyaluronate, 47.5 wt.% chitosan, and 47.5 wt.% bioglass treated at 550 °C/3 h (C–H5–P5, C–H5–P5Zn2, C–H5–P5Sr2) or at 650 °C/10 h (C–H5–P5d, C–H5–P5Zn2d, C–H5–P5Sr2d) and containing 10 wt.% sodium hyaluronate, 45 wt.% chitosan, and 45 wt.% bioglass treated at 550 °C/3 h (C–H10–P5, C–H10–P5Zn2, C–H10–P5Sr2) or at 650 °C/10 h (C–H10–P5d, C–H10–P5Sr2d).

#### 4.7.1. Extraction Procedure

The mass of the biomaterial used in the biological test was normalised to the mass of the biocomposites (C–H5 or C-H10, i.e., containing 5 wt.% of sodium hyaluronate, 47.5 wt.% chitosan, and 47.5 wt.% bioglass or containing 10 wt.% of sodium hyaluronate, 45 wt.% chitosan, and 45 wt.% bioglass, respectively) by taking into account the appropriate percentage of bioglass and chitosan/hyaluronic acid in the prepared controls. The concentration of the composite extract was 2 mg/mL. The study was carried out using de novo prepared extracts. To prepare extracts for biomaterials, RPMI-1640 with antibiotics was added according to the indicated proportion. During extraction, the samples were incubated at 37 °C and 5% CO_2_ for 24 h. After extraction was complete, the culture fluid was collected and centrifuged at 200 rpm for 15 min.

#### 4.7.2. Indirect Contact Cytotoxicity Test

A culture of A549 cells with a density of 3 × 10^4^/mL was established in a 96-well plate in RPMI-1640 with the addition of 2% fetal calf serum (FCS) and antibiotics and incubated at 37 °C in an atmosphere of 5% CO_2_ for 24 h. After incubation time, the culture supernatant was removed, and 0.2 mL of the extract obtained was added. Subsequently, the cultures were incubated at 37 °C and 5% CO_2_ for 48 h. Quantitative and morphological changes caused by the extracts tested were evaluated using an inverted optical microscope.

#### 4.7.3. Colorimetric MTT Assay

An amount of 25 µL of MTT stock solution (5 mg/mL) was added to each well, and the plates were incubated for additional 3 h in a cell culture incubator at 37 °C and 5% CO_2_. Then, 100 µL of extraction buffer (50% DMF with 20% SDS, pH 4.7) was added. After 15 h of incubation, the optical density (OD) was measured at 550 nm with a reference wavelength of 630 nm using a Dynatech 5000 spectrophotometer (Dynatech Laboratories Inc., Alexandria, VA, USA).

### 4.8. Whole Blood Culture Test and Proinflammatory Cytokine Production

#### 4.8.1. Origin of Blood

The study was approved by the Ethics Committee (Institute of Immunology and Experimental Therapy, Polish Academy of Sciences in Wrocław—approval no. KB-1/2021) and was carried out in accordance with the Declaration of Helsinki. Informed consent was obtained from all subjects/volunteers involved in the study. Complete peripheral blood was collected from volunteers using heparinised syringe systems at the Blood Donation Station. The donors were healthy men aged 19 to 23 years, diagnosed as free of viral and bacterial infections, inflammation, and allergic diseases, not treated with any antiplatelet, anticoagulant, or anti-inflammatory drugs or antibiotics within 14 days before blood donation.

#### 4.8.2. Whole Blood Culture Tests

The test used heparinised blood from healthy donors diluted with RPMI-1640 medium with the addition of antibiotics. The extracts from the composite samples and their components, prepared according to a method described in Section 4.7.1, were introduced to the cultured cells in a 1:9 ratio. The extracts were tested in a blood cell culture without or with the addition of 1 µg/mL LPS. Additionally, the test included spontaneous control and control with the addition of LPS. The culture was incubated at 37 °C for 20 h in an atmosphere of 5% CO_2_. After incubation, the cells were centrifuged, and supernatants were collected and frozen at −80 °C.

#### 4.8.3. Proinflammatory Cytokine Determination

Cytokine determination in the preserved supernatants was performed using the ELISA kit, TNF-α and IL-6, according to the attached protocol. Cytokine levels in the supernatants were determined using Biolegend ELISA kits for TNF-α and IL-6 according to the manufacturer’s protocols. The optical density was measured using a Multiskan RC spectrophotometric reader (Thermo Labsystems, Waltham, MA, USA) at λ = 450 nm.

### 4.9. Cytocompatibility Test with MG63 Cells

#### 4.9.1. Sample Preparation and Cell Culture Conditions of the Samples

For the direct in vitro test, the samples were prepared in the form of discs. Glass samples with a diameter of 6 mm and a height of ca. 3 mm were prepared using a PYTE model uniaxial press at a pressure of 5 MPa. Biocomposite hydrogel–bioglass samples with a diameter of 12 mm and a height of 2 mm were prepared by casting in PTFE moulds.

The MG-63 osteoblast-like cells (European Collection of Cell Cultures, Salisbury) were cultured in modified Eagle medium (MEM, PAN BIOTECH, Aidenbach, Germany) supplemented with 10% foetal bovine serum (FBS) (Biowest, Nuaillé, France), 5% amino acids and sodium pyruvate, and 1% penicillium/streptomycin (all PAN BIOTECH, Germany). A total of 2 × 10^4^ cells per sample were seeded and cultured at 37 °C, 5% CO_2_, with increased humidity. The surface of tissue culture polystyrene (TCPS, 24-well culture plate, Nunclon) was used as a control.

#### 4.9.2. AlamarBlue and Live–Dead Tests

On days 1, 3, and 7 of cell viability, proliferation, and morphology were tested with the use of AlamarBlue^®^ (resazurin-based, Sigma-Aldrich, Taufkirchen Germany) and live/dead staining protocols. Briefly, bioglass and composite samples were transferred to the wells of a new plate, and 1 mL of a 5% AlamarBlue solution in medium was added to each well and incubated for 2.5 h. Subsequently, 100 µL of the solution was collected from each well and transferred to a black 96-well plate, and fluorescence was measured (λ_ex_ = 544 and λ_em_ = 590 nm, FluoStar Omega, BMG Labtech, Ortenberg, Germany). The percentage of resazurin reduction was calculated using the following formula:

% *rezasurin reduction* = (*S_x_* − *S_blank_*)/(*S_reduced_* − *S_blank_*) · 100%

where *S_x_* is the fluorescence of the sample, *S_blank_* is the fluorescence of the MEM with 5% AlamarBlue reagent, without cells (0 resazurin % reduction), and *S_reduced_* is the fluorescence of the completely reduced MEM with 5% AlamarBlue reagent autoclaved for 15 min at 121 °C (100% resazurin reduction).

Because the samples and the control TCPS had different areas (bioglasses—28 mm^2^, scaffolds—113 mm^2^, TCPS—191 mm^2^), the results were normalised and expressed as % resazurin reduction per cm^2^ of the respective sample. Subsequently, the medium was exchanged, and the cells were cultured for 3 and 7 days.

Live/dead staining was performed in a propidium iodide and calcein AM mixture (both 1 mg/mL, Sigma-Aldrich, Germany), which was added to the wells and subsequently incubated for 20 min in the dark. Afterwards, the samples were observed with the inverted fluorescence microscope AxioVert 40 (Carl Zeiss, Oberkochen, Germany, equipped with metal halide illuminator).

## 5. Statistics

All experiments were carried out in triplicate, and the data are expressed as the mean ± standard deviation of three or more independent experiments. Statistical significance was determined using a one-way analysis of variance (ANOVA) with the LSD Fisher post hoc test. The analyses were calculated using OriginPro 2020 (OriginLab Corp., Northampton, MA, USA).

## Figures and Tables

**Table 1:** The amount of Zn^2+^ and Sr^2+^ released from composites into deionised water after 24 h or 7 days of incubation at 37 °C.

Symbol of Sample	Incubation Period	Zn^2+^ Ions (mg/L)	Sr^2+^ Ions (mg/L)
C–H10–P5Zn2	24 h	0.34 ± 0.04	^bdl^
C–H10–P5Zn2d	24 h	0.33 ± 0.04	^bdl^
C–H10–P5Sr2	24 h	^bdl^	0.86 ± 0.10
C–H10–P5Sr2d	24 h	^bdl^	1.30 ± 0.15
C–H10–P5Zn2	7 days	0.24 ± 0.03	^bdl^
C–H10–P5Zn2d	7 days	0.20 ± 0.02	^bdl^
C–H10–P5Sr2	7 days	^bdl^	1.94 ± 0.23
C–H10–P5Sr2d	7 days	^bdl^	2.45 ± 0.29

^bdl^—below detection limit.

**Table 2:** The thermal treatment parameters and compositions and symbols of bioglasses after heating.

The parameters of thermal treatment	Bioglass composition		
	SiO_2_ 70 wt.%	SiO_2_ 70 wt.%	SiO_2_ 70 wt.%
	P_2_O_5_ 5 wt.%	P_2_O_5_ 5 wt.%	P_2_O_5_ 5 wt.%
	CaO 25 wt.%	CaO 23 wt.%	CaO 23 wt.%
		ZnO 2 wt.%	SrO 2 wt.%
550 °C/3 h	P5	P5Zn2	P5Sr2
(d): 650 °C/10 h	P5d	P5Zn2d	P5Sr2d

**Table 3:** The symbols of chitosan/sodium hyaluronate composites.

Thermal treatment parameters of bioglasses	Composition of bioglass used for composites preparation		
	SiO_2_ 70 wt.%	SiO_2_ 70 wt.%	SiO_2_ 70 wt.%
	P_2_O_5_ 5 wt.%	P_2_O_5_ 5 wt.%	P_2_O_5_ 5 wt.%
	CaO 25 wt.%	CaO 23 wt.%	CaO 23 wt.%
		ZnO 2 wt.%	SrO 2 wt.%
550 °C/3 h	C–H2–P5	C–H2–P5Zn2	C–H2–P5Sr2
	C–H5–P5	C–H5–P5Zn2	C–H5–P5Sr2
	C–H10–P5	C–H10–P5Zn2	C–H10-P5Sr2
(d): 650 °C/10 h	C–H2–P5d	C–H2–P5Zn2d	C–H2–P5Sr2d
	C–H5–P5d	C–H5–P5Zn2d	C–H5–P5Sr2d
	C–H10–P5d	C–H10–P5Zn2d	C–H10–P5Sr2d

## Data Availability

The data generated during this study are available at the ŁUKASIEWICZ Research Network, Institute of Ceramics and Building Materials, Biomaterials Research Group, Postępu 9, Warsaw, 02-676, Poland, and biological data are available at the Department of Biomaterials and Composites, Faculty of Materials Science and Ceramics, AGH University of Science and Technology, Al. Mickiewicza 30, Krakow, 30-059, Poland and at the Hirszfeld Institute of Immunology and Experimental Therapy, Laboratory of Immunobiology, Polish Academy of Sciences, R. Weigla Str. 12, Wroclaw, 53-114, Poland and are available from the corresponding authors upon request.
